# Connecticut Valley Dental Association

**Published:** 1879-01

**Authors:** 


### Proceedings of Societies.

### CONNECTICUT VALLEY DENTAL ASSOCIATION.

The annual meeting of the Connecticut State Dental Society was held June 14, in Springfield, Mass. The session opened with about forty members in attendance. After organization the reports of officers was made and approved. After this the election of officers for the ensuing year was declared to be in order, which being had, the following were found to be elected, viz:

President, Dr. C. A. Bracket, of Newport, R. I.
First Vice President, Dr. C. S. Hurlburt, of Springfield.
Second Vice President, Dr. C. Fones, of Bridgeport, Ct.
Secretary, Dr. C. T. Stockwell, of Springfield, Mass.
Treasurer, Dr. N. Morgan, of Springfield, Mass.

The retiring president, Dr. L. C. Taylor, now delivered his annual address, on the subject, "What constitutes a good dentist." It was full of good thoughts and suggestions.

Dr. E. S. Niles, of Harvard Dental School, read a paper on the "Inervation of the salivary glands."

"Toothache, causes, diagnosis and treatment," was discussed at considerable length, especially in respect to the influence of malaria as a cause or an excitant.

The cases in which this influence is present, are amenible to anti-periodic treatment, at least, this seemed to be the view of those who gave an opinion on the subject.

"The relation of the profession to the public," was the subject of quite a spirited discussion, the general conclusion of which was that physical as well as mental culture is a necessity for him who will best serve those placed in his charge, and also that the moral and sympathetic nature should be cultivated, and that gentlemanly deportment and propriety should always characterize the true professional man.

A very pleasant turn in the proceeding was made just at this point, it was especially so to one of the officers. Prof. L. D. Shephard, on behalf of the society, presented Dr. C. T. Stockwell, the secretary, with a purse of forty dollars in gold, as a testimonial of esteem for his voluntary and laborious work for the society.

The subject of "Oral Surgery," was brought under discussion, the chief participaters being Prof. Shepard and Dr. Searl, the former taking the ground that it properly embraces the treatment of, and operations upon the teeth and associate parts. The latter considered oral surgery and dentistry as two distinct specialties, defining the former as curing, by manual operation, diseases of the mouth and associate parts.

He believed dentistry proper was excluded when treating disease of the alveolar process, and anything beyond. Though the dentists of the present are not qualified for oral surgery, this will undoubtedly be taught in the future with dentistry.

Prof. Shepard strongly urged the extension of the curriculum of dental education, this seemed to receive the hearty endorsement of the members present.

The next meeting of the association is to be held in June next, in Boston, to which all the dental societies in New England are invited.

